# Effects of canola and corn oil mimetic on Jurkat cells

**DOI:** 10.1186/1476-511X-10-90

**Published:** 2011-06-01

**Authors:** Gabriela Ion, Kayla Fazio, Juliana A Akinsete, W Elaine Hardman

**Affiliations:** 1 Department of Biochemistry and Microbiology, Marshall University School of Medicine, Huntington, WV, USA; 2 Bluefield State College, Bluefield, WV, USA

**Keywords:** Lymphocytes, Canola oil mimetic, Corn oil mimetic, Apoptosis, Inflammation

## Abstract

**Background:**

The Western diet is high in omega-6 fatty acids and low in omega-3 fatty acids. Canola oil contains a healthier omega 3 to omega 6 ratio than corn oil. Jurkat T leukemia cells were treated with free fatty acids mixtures in ratios mimicking that found in commercially available canola oil (7% α-linolenic, 30% linoleic, 54% oleic) or corn oil (59% linoleic, 24% oleic) to determine the cell survival or cell death and changes in expression levels of inflammatory cytokines and receptors following oil treatment.

**Methods:**

Fatty acid uptake was assessed by gas chromatography. Cell survival and cell death were evaluated by cell cycle analyses, propidium-iodide staining, trypan blue exclusion and phosphatidylserine externalization. mRNA levels of inflammatory cytokines and receptors were assessed by RT-PCR.

**Results:**

There was a significant difference in the lipid profiles of the cells after treatment. Differential action of the oils on inflammatory molecules, following treatment at non-cytotoxic levels, indicated that canola oil mimetic was anti-inflammatory whereas corn oil mimetic was pro-inflammatory.

**Significance:**

These results indicate that use of canola oil in the diet instead of corn oil might be beneficial for diseases promoted by inflammation.

## Background

The ratio of omega-3 to omega-6 in the average western diet is heavily weighted in favor of omega-6 [1]. When tested as single fatty acids, omega 6 fatty acids tend to be pro-inflammatory but omega-3 fatty acids tend to be anti-inflammatory. Therefore, omega-3 deficiencies have been implicated in inflammatory diseases, cancer, cardiovascular diseases, dyslipidaemia and metabolic syndrome [1,2].

The human diet is very complex and foods provide a mixture of fatty acids in different ratios not just one single fatty acid at a time. Food is the source of two essential fatty acids, linoleic (omega-6) and α-linolenic acid (omega-3), which cannot be synthesized *de novo *in animal cells and, therefore, must be obtained from the diet. A good dietary source of omega-3 with an omega-6 to omega-3 ratio of 3:1 is canola oil. We hypothesize that consuming canola oil in the diet instead of corn oil could decrease pro-inflammatory stimuli.

There is a lack of data aimed at exploring the effect of complex combinations of food fats in *in vitro *models. In general, many *in vitro *models focus on only single fatty acids at different concentrations [3-6]. Therefore, to be more relevant to human health, it might be beneficial to consider an experimental design closer to the ratios of the components found in the food which might be consumed.

There is a body of evidence demonstrating that fatty acids affect T lymphocyte functions. In vitro and in vivo studies have shown that fatty acids modulate cytokine release, proliferation, cell death, activation by antigens, surface proteins expression and signaling proteins [7-14]. Single free fatty acids have been shown to induce cell death when used at various concentrations in different cellular models [4,5,8,15,16]. To study the pro- or anti-inflammatory effects of fatty acids combinations on cytokine production by lymphocytes it is important to explore the effects of fatty acids at non cytotoxic doses. These data would be more relevant to a typical diet where food ingested does not have a cytotoxic effect and could demonstrate alterations in inflammatory cytokines.

In spite of the well-recognized beneficial effects of omega-3 fatty acids for human health, there is a lack of data regarding the effect of canola oil, a common food source rich in α-linolenic acid (omega-3 fatty acid) versus corn oil rich in linoleic acid (omega-6 fatty acid), on lymphocytes. In this study, Jurkat T leukemia cells were treated with free fatty acids mixtures in ratios mimicking that found in commercially available canola oil (7% α-linolenic, 30% linoleic, 54% oleic) or corn oil (59% linoleic, 24% oleic) at non cytotoxic dose to determine changes in expression levels of inflammatory cytokines and receptors following oil treatment.

## Methods

### Reagents

The following reagents were used: propidium iodide, Tri-Reagent, 2-propanol, 1 bromo-3-chloro propane, RNase A, ethanol, 3-sodium citrate, butylated hydroxytoluene (BHT) from Sigma-Aldrich; α-linolenic acid (Cayman Chemical Company), linoleic acid and oleic acid (MP-Biomedicals, LLC); Triton-X100 (IBI Shelton Scientific, Inc.); Chloroform and Hexane (Honeywell, Burdick & Jackson^™^), Methanol (Fisher Scientific), Isooctane (EMD).

### Cell Lines

Jurkat, Clone E6-1 cells (gift from Dr. Pyali Dasgupta, MU) were maintained in 10% FBS (Hyclone) in RPMI-1640 (ATCC) supplemented with 100 units/ml penicillin and 0.1 mg/ml streptomycin (Sigma-Aldrich). The cells were kept in a humidified atmosphere, at 37°C, containing 5% CO_2_. The cells were seeded at a cell density of 3 × 10^5 ^per ml for all experimental designs.

### Fatty acid treatment

The free fatty acids, in ratios mimicking that found in commercially available canola oil (7% α-linolenic, 30% linoleic, 54% oleic) or corn oil (59% linoleic, 24% oleic) were dissolved in ethanol. Cells were treated with an oil concentration of 75 μM, 100 μM or 150 μM for 48 or 72 hours. The final concentration of ethanol in culture media did not exceed 0.15%.

### Cell viability and membrane integrity

Cell viability and membrane integrity were assessed by Trypan Blue exclusion and propidium iodide staining, respectively. After treatment, the cells were washed with PBS and stained with propidium iodide (20 μg/ml) for 15 min in the dark, at room temperature. The cells were analyzed on a FACSAria flow cytometer (Becton Dickinson) using DIVA software (Becton Dickinson) and the propidium iodide positive population was evaluated. The Trypan Blue exclusion assay was used to determine cell viability, and the live cells (negative for staining) and dead cells (positive for staining) were enumerated using a hemocytometer.

### Annexin V labeling

Jurkat cells were treated as indicated, then washed twice with PBS and resuspended in Annexin V binding buffer (0.01 M HEPES, 0.14 M NaCl and 2.5 mM CaCl_2_). Annexin V-Pacific Blue™ conjugate (Invitorgen, Molecular Probes) and propidium iodide (20 μg/ml) were added to the cells for 15 min in the dark, at room temperature. Cells were analyzed on a FACSAria flow cytometer using DIVA software and the Annexin V positive/propidium iodide negative population was considered early apoptotic.

### Cell cycle and DNA fragmentation

Treated cells were subjected to DNA content analysis. Briefly, the cells were harvested and washed two times with PBS and fixed with cold 70% ethanol for at least 24 hours. The ethanol was removed and followed by two PBS washes. Cells were stained in the following solution: PBS supplemented with 0.1% Triton X-100, 0.1% Na_3_-citrate, 30 μg/ml RNase and 20 μg/ml propidium iodide. After incubation in the dark for 30 minutes at room temperature the cells were analyzed on a FACSAria flow cytometer. DNA fragmentation was determined by cell cycle analysis using DIVA software.

### Gas chromatography

The fatty acid composition of treated cells was analyzed by gas chromatography. After treatment, cells were washed four times in PBS then homogenized in distilled water containing 0.1% BHT to prevent fatty acid oxidation. Lipids were extracted with chloroform/methanol, and the fatty acids were methylated followed by separation and identification using gas chromatography. Briefly, gas chromatography was performed using a PerkinElmer Clarus 500 Gas Chromatograph (Shelton, CT) with a Elite-WAX Polyethylene Glycol Capillary Column (Length: 30 m, Inner Diameter: 0.53 mm), at 220°C for 100 min with a helium carrier gas flow rate of 2 ml/min. A fatty acid methyl ester standard (Nu-Chek-Prep, Elysian, MN) GLC #704, which contains 10 fatty acids (methyl esters of stearate, oleate, linoleate, alpha linolenate, gamma liniolenate, homogamma linolenate, arachidonate, eicosapentaenoate, docosapentaenoate, and docosahexaenoate) was used for peak identification. The fatty acid methyl esters were reported as the percent of the total methylated fatty acids (area under the curve).

### Gene expression assay

Human Inflammatory Cytokines and Receptors RT^2 ^*Profiler*^™ ^PCR Array, RT2 First Strand Kit and SuperArray RT2 qPCR Master Mix (SuperArray Bioscience Corporation, Frederick, MD) were used to analyze the expression of a panel of genes in cells treated at 75 μM oil concentration for 72 hours. After treatment, cells were homogenized in Tri Reagent following the protocol of the manufacturer to isolate the RNA. RNA quality control was performed for all samples. The gene expression assay followed the protocol provided by SuperArray. The relative fold differences in gene expression and statistical analyses were calculated on SuperArray software.

## Results

### Lipid composition of treated cells

**Figure 1:**
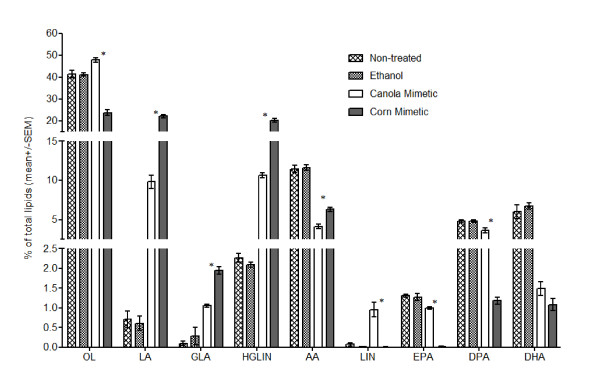
**Lipid composition of Jurkat cells treated with 150 μM canola or corn oil mimetics**. The values are presented as mean +/- SEM of four samples. * p < 0.005 for comparison between the canola versus corn oil mimetic treatment by t-test. OL--oleic acid; LIN--α-linolenic acid; LA--linoleic acid; EPA--eicosapentaenoic acid; GLA--gamma linolenic acid; DPA--docosapentaenoic acid; HGLIN--homo-gamma linolenic acid; DHA--docosahexaenoic acid; AA--arachidonic acid.

Gas chromatography was performed to investigate whether the cells were able to uptake the canola and corn oil mimetic. Jurkat cells were treated with 150 μM canola or corn oil mimetic for 72 hours. When comparing the canola oil mimetic treatment with the corn oil mimetic treatment, the canola oil mimetic treated cells had significantly more α-linolenic (LIN) acid and more of the biosynthetic omega-3 fatty acids products (EPA, DPA) (n = 4, p < 0.005) (Figure 1). The corn oil mimetic treated cells showed increased levels of linoleic acid (LA) and the biosynthetic omega-6 fatty acids products (GLA, HGLIN, AA) at a higher fraction than canola oil mimetic (n = 4, p < 0.005) (Figure 1).

Ethanol was used as a carrier for the fatty acids and addition to cell culture did not induce any change in lipid content compared to non-treated cells. As was expected, comparison of either fatty acid treatment (canola or corn oil mimetic) to ethanol and non-treated controls demonstrates a change in the lipid content. Both the canola and corn oil mimetic increased the percentage of linoleic acid (an omega-6 fatty acid) when compared to ethanol and non-treated cells. Although the canola mimetic contains more omega-3 fatty acids than corn oil mimetic, it is essential to note that it also contains a percentage (30%) of linoleic acid, although less than the corn oil mimetic (59%). Therefore, the increase in linoleic acid for both treatments is expected. In contrast, when comparing the two oil mimetics, it is apparent that the canola oil mimetic (containing just 7% α-linolenic acid) is generating a higher fraction of omega-3 fatty acids than the corn oil mimetic. Despite the comparable percentages of EPA or DPA between controls (non treated and ethanol treated cells) and the canola oil mimetic, noteworthy is the decrease in omega-3 fatty acids in the presence of the corn oil mimetic.

### Membrane Integrity

**Figure 2:**
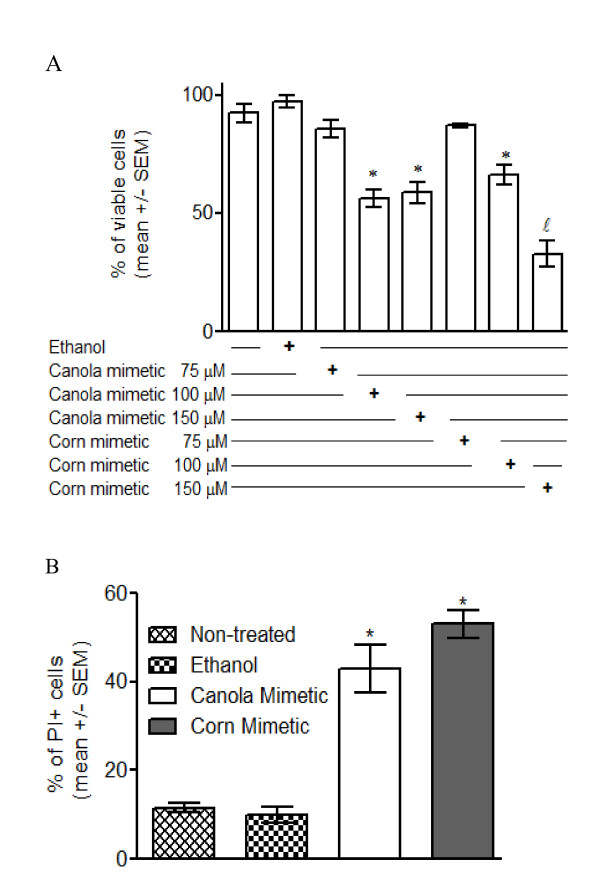
**Membrane integrity of Jurkat cells treated with canola and corn oil mimetics**. **A**. Percentage of viable cells, treated for 72 hours with 75, 100 and 150 μM oil mimetics, showed by Trypan Blue exclusion. The values are presented as mean +/- SEM of six samples.* p < 0.05 by Newman-Keuls multiple comparison test, when compared to controls (non-treated and ethanol treated cells); *ℓ *p < 0.05 by Newman-Keuls multiple comparison test, when compared to the controls and all other treatments. **B**. percentage of cells losing the membrane integrity, treated for 48 hours with 150 μM oil mimetics, showed by propidium-iodide staining * p < 0.05 by Newman-Keuls multiple comparison test n = 3 for non-treated, n = 4 for all other samples.

Trypan Blue exclusion (Figure 2A) showed that treatment with 100 and 150 μM canola or corn oil mimetic treatment for 72 hours significantly decreased the percentage of viable cells when compared to the controls (non-treated and ethanol treated cells) (n = 6, p < 0.05). Among the treatments, 150 μM corn oil mimetic showed the highest decrease of cell viability by Trypan blue (n = 6, p < 0.05) (Figure 2A). Propidium-iodide staining (Figure 2B) showed a significant increase in the percentage of cells that lost of membrane integrity in the treated cells (150 μM canola or corn oil mimetic for 48 hours) when compared to the controls (non-treated and ethanol treated cell) (n = 3 for non-treated, n = 4 for all other samples, p < 0.05).

### Apoptosis

**Figure 3:**
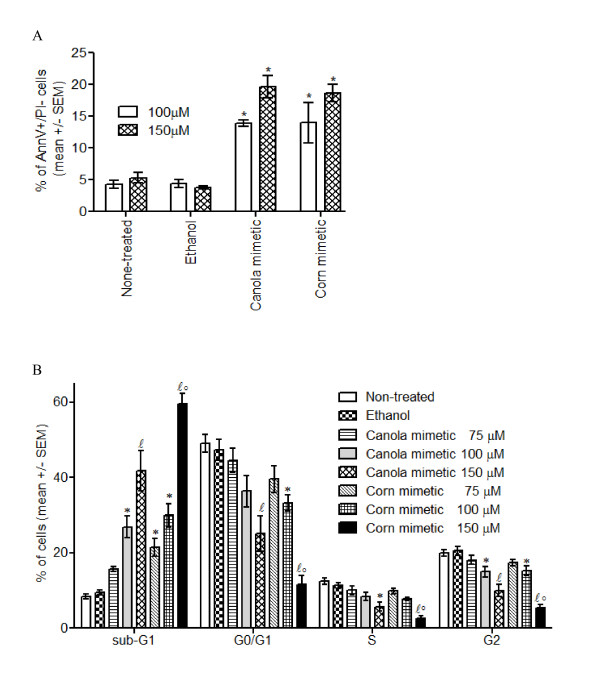
**Apoptosis **A**. Phosphatidylserine exposure in cells treated at 100 μM and 150 μM for 48 hours**. The values are presented as mean +/- SEM of three samples for 150 μM non-treated and four samples for all other treatments and controls. * p < 0.05 by Newman-Keuls multiple comparison test. **B**. Cell cycle analysis of cells treated with 75, 100 or 150 μM oils for 72 hours. The values are presented as mean +/- SEM of six samples. * p < 0.05 by Newman-Keuls multiple comparison test, when compared to controls (non-treated and ethanol treated cells); *ℓ *p < 0.05 by Newman-Keuls multiple comparison test, when compared to the controls and 75 and 100 μM oil treatments; °p< 0.05 by Newman-Keuls multiple comparison test, when compared to 150 μM canola oil mimetic treatments.

The loss of membrane asymmetry and the exposure of phosphatidylserine on the outer surface of the cell membrane as an early apoptotic marker was detected with Pacific Blue labeled AnnexinV. Jurkat cells treated with canola or corn oil mimetic at 100 μM and 150 μM for 48 hours showed a significant increase in Annexin V^+^/propidium iodide^- ^population compared to the controls (non-treated and ethanol treated cells) (n = 3 for 150 μM non-treated, n = 4 for all other samples, p < 0.05) (Figure 3A).

### DNA fragmentation

The degradation of the nuclear DNA as a late apoptotic marker was assessed by the formation of the 'sub-G1' population [17]. Cell cycle analysis of Jurkat cells treated with 75, 100 or 150 μM canola or corn oil mimetic for 72 hours was evaluated (Figure 3B). When comparing the treatments to the controls (non-treated and ethanol treated cells), there was a significant increase in the 'sub-G1' population for the following treatments: 100 and 150 μM canola oil mimetic and 75, 100 and 150 μM corn oil mimetic (n = 6, p < 0.05) (Figure 3B). When comparing the 150 μM corn oil mimetic treatment with the 150 μM canola oil mimetic treatment, the 'sub-G1' population was significantly higher in the corn oil mimetic treated cells.

The exposure of early apoptotic marker phosphatidylserine at 48 hours followed by loss of membrane integrity and DNA fragmentation at 72 hours indicates that treatment with ≥ 100 μM canola or corn oil mimetic induced apoptosis in Jurkat cells.

### Cell cycle

There was a significant decrease in the percentage of Jurkat cells in the G0/G1 phase for cells treated with 100 μM corn oil mimetic compared to the controls (non-treated and ethanol treated cells) (n = 6, p < 0.05) (Figure 3B). There was a significant decrease in the percentage of Jurkat cells in the G0/G1 phase for cells treated with 150 μM canola or corn oil mimetic when compared to the controls (non-treated and ethanol treated cells) or to the other treatments (n = 6, p < 0.05) (Figure 3B). Moreover, when comparing 150 μM corn oil mimetic to 150 μM canola oil mimetic treatment there was a statistically significant difference in the G0/G1 phase between the treatments.

There was a significant decrease in the percentage of cells in the S phase of both 150 μM canola and corn oil mimetic treated cells when compared to the controls (non-treated and ethanol treated cells) (n = 6, p < 0.05) (Figure 3B). The percentage of cells in the S phase was significantly decreased by 150 μM corn oil mimetic when compared to all other treatments.

The percentage of cells in G2 exhibited a significant decrease for 100 and 150 μM oil treatment when compared to the controls. Cells treated with 150 μM oil mimetic exhibited a significant decreased fraction in the G2 phase when compared to 75 and 100 μM canola or corn oil mimetic. Also, there was a significant difference in the G2 phase fraction between corn and canola oil mimetic at 150 μM concentration.

### Inflammatory cytokines and receptors

The highest concentration of oil not inducing significant changes in the cell cycle of Jurkat cells was 75 μM. This dose was utilized to investigate the effect of canola and corn oil mimetic on expression of inflammatory cytokines and receptors. Jurkat cells treated with 75 μM canola oil mimetic for 72 hours showed a 3.46 fold up-regulation (n = 3, p = 0.0193) for CCL5 (RANTES), compared to corn oil mimetic treated cells (Table 1). Moreover, gene expression analysis following canola oil mimetic treatment showed a trend towards down-regulating expression of CCL11, CARD18, IL8 and IL8RB when compared to the controls (non-treated and ethanol treated cells). Whereas, corn oil mimetic treatment showed a trend towards up-regulating the expression for the same genes (CCL11, CARD18, IL8, IL8RB) as compared to the controls. To better assess the differences in the gene profiling as a response to the oil mimetic treatments, the oil treatment groups were compared. Table 1 shows a significant down-regulation of gene expression in canola oil mimetic treated cells when compared to corn oil mimetic treated cells. A low oil concentration (75 μM) did not have a significant effect on cell cycle but was able to induce differences in gene expression.

**Table 1:** Gene expression in Jurkat cells treated with canola or corn oil mimetic

Gene	canola mimetic vs controls		corn mimetic vs controls		canola mimetic vs corn mimetic	
	
	Fold difference	p value	Fold difference	p value	Fold difference	p value
CCL11	-1.30	0.2172	1.30	0.2112	**-1.69**	**0.0185**

CCL18	-1.30	0.2172	1.76	0.2705	-2.29	0.2413

CCL5	1.61	0.2205	-2.15	0.0959	**3.46**	**0.0193**

CCR2	1.06	0.8768	-1.94	0.2205	2.05	0.3665

CXCL11	**-2.07**	**0.0515**	-1.43	0.2968	-1.45	0.1039

CARD18	-1.48	0.1408	1.30	0.3460	**-1.93**	**0.0364**

IL5	1.54	0.3790	-2.08	0.5255	3.20	0.4556

IL8	-1.57	0.1646	1.38	0.3903	-2.17	0.0687

IL8RB	-1.51	0.1071	1.30	0.2542	**-1.97**	**0.0025**

Jurkat cells were treated with oils at 75 μM for 72 hours. The values represent the fold change in canola mimetic treated cells versus controls; corn oil mimetic treated cells versus controls; canola oil mimetic versus corn oil mimetic treated cells. n = 3 for canola or corn oil mimetic treated cells; n = 5 for controls (non-treated and ethanol treated cells pooled together). (+) = increased fold change; (-) = decreased fold change

## Discussion

It is widely accepted that free fatty acids can induce cell death in *in vitro *models [4,8,15,18]. Free fatty acid mixtures in ratios mimicking that found in commercially available canola oil (7% α-linolenic, 30% linoleic, 54% oleic) or corn oil (59% linoleic, 24% oleic) had a cytotoxic effect on Jurkat T leukemia cells at high concentration (≥ 100 μM). Even though both treatments were cytotoxic one hundred fifty micromolar canola or corn oil mimetic treated cells resulted in different lipid compositions and significant differences in cell cycle and cell death response indicating that the treatments were doing more than just killing cells. Corn oil mimetic treated Jurkat cells had a significantly higher uptake of linoleic acid followed by synthesis of more longer chain omega-6 fatty acids (gamma linolenic, homo-gamma linolenic, arachidonic acid) than canola oil mimetic treated cells. Canola oil mimetic treated cells had a significantly higher uptake of α-linolenic acid and were able to synthesize more of the longer chain omega-3 fatty acids (eicosapentaenoic acid, docosapentaenoic acid, docosahexaenoic acid) than corn oil mimetic treated cells.

Both oil mimetic treatments at a concentration ≥ 100 μM increased the DNA fragmentation ('sub-G1 population'). The DNA fragmentation was associated with a decreased in the percentage of cells in the other phases of cell cycle. Previous studies using individual fatty acids showed that linoleic acid was cytotoxic at 100 μM and α-linolenic acid had an anti-proliferative effect at 60 μM [11]. Cury-Boaventura et al. [8] showed that 50 and 100 μM linoleic acid induced phosphatidylserine exposure, an early marker for apoptosis, on human lymphocytes. The authors suggested mitochondrial depolarization and ROS production as a mechanism for cell death induced by 200 μM linoleic acid. ROS represent key molecules involved in multiple cellular functions like cell adhesion, apoptosis, regulation of immune responses [19]. On Jurkat cells 130 μM linoleic acid or 60 μM α-linolenic acid had a prooxidant-induced antiproliferatve effect that was negatively correlated with caspase 3 activation [11]. In addition, the pro-apoptotic activity of α-linolenic acid has been associated with up regulation of Bax expression and cytochrome c translocation [18]. In support of these previous studies, the present study demonstrates that canola and corn oil mimetic induced apoptosis and significant changes in cell cycle at a concentration of 100 μM. Further investigations are required to establish the mechanism involved in linoleic and α-linolenic fatty acids modulation of cell cycle progression. Many studies have used single long chain omega 3 or 6 fatty acids to modulate cell cycle progression in different cancer cell lines [20-23]. For example, arachidonic acid increased expression of cyclin D1 mRNA and the percentage of cells in S phase [20]. Docosahexaenoic acid reduced cyclin D1, E, and A-associated kinase activity and prevented the entry of cells in S phase [21]. Eicosapentaenoic acid inhibited synthesis and expression of cyclin D1 and E and blocked cell cycle in G1 [22]. *Trans*-10, *cis*-12 conjugated linoleic acid increased the levels of p21^cip1/waf1 ^and blocked the cells in G0/G1 [23]. However, there is a lack of data regarding the effect of the two essential fatty acids, linoleic and α-linolenic acid present in the most commonly cook oils, corn and canola oil, respectively.

The association between inflammation and cancer is thought to be a critical component for cancer development [24]. The polyunsaturated fatty acids (n-3, n-6) are responsible for the production of families of anti- and pro-inflammmatory bioactive lipid mediators [25]. *In vivo *studies showed that omega 3 fatty acids decreased chemoattractant protein-1 (MCP-1), interleukin (IL)-6, interferon (IFN)-gamma mRNA expression [26], and TNF-α level [27]. *In vitro *linoleic acid and α-linolenic acid inhibited IL-2 production [11]. In this work, a low oil concentration (75 μM) did not have a significant effect on cell cycle but was able to induce differences in gene expression. One such gene, CCL5 (Regulated upon Activation, Normal T-cell Expressed, and Secreted, abbreviated RANTES) has a dual role regarding tumorigenesis. CCL5 can mediate tumor cell survival, cell growth and metastasis in a number of malignances [28-31]. CCL5 is also proposed as a natural adjuvant to boost anti-tumor immunity [32]. In our experimental design, canola oil mimetic increased CCL5 expression compared to corn oil mimetic treatment. Moreover, the proinflammatory molecules, IL8 (interleukin8), IL8RB (interleukin8 receptor, beta known as CXCR2), CARD18 (caspase recruitment domain family, member 18; ICEBERG) and CCL11 (chemokine (C-C motif) ligand 11) were slightly down-regulated in canola oil mimetic Jurkat treated cells compared to slightly up-regulated in corn oil mimetic treatment. IL-8, a chemotactic factor for leukocytes, has been shown to contribute to human cancer progression through its potential functions as a mitogenic and angiogenic factor [33]. CARD18 (ICEBERG), induced by pro-inflammatory stimuli, inhibits generation of IL-1β by interacting with caspase-1 and preventing its association with RIP2 [34]. CCL11 (eotaxin-1) displays chemotactic activity for eosinophils [35] and is a key player in the angiogenic cascade [36]. Taken together, suppression of these chemokines would be expected to slow cancer progression.

## Conclusion

This study was designed to explore the effects of oil mimetics in ratios found in two common cooking oils (canola and corn) on Jurkat T leukemia cells. At high concentrations (100 and 150 μM) both types of oils induced apoptosis. At a non-toxic dose (75 μM) the different oil mimetics displayed differences in their action on pro-inflammatory molecules with canola oil being anti-inflammatory whereas corn oil was pro- inflammatory. Findings from this study emphasize the need to investigate the effect of dietary fat within complex mixtures at non-cytotoxic doses when evaluating the inflammatory response. Oil mimetic could be enough to induce differences in fatty acid and immune modulator profiles. This is critical in regards to the importance of examining conventional diet sources in human health and disease. Oil mixtures are more physiologically relevant than single fatty acids since humans must consume both omega-3 and omega-6 fatty acids. In this respect canola oil may have a more favorable fatty acid profile for decreasing the chance of inflammation that is promotional for development of chronic diseases.

## Competing interests

The authors declare that they have no competing interests.

## Authors' contributions

All authors have read and approve the final manuscript. GI designed the study, analyzed, interpreted the data, and drafted the manuscript. KF carried out the apoptosis assays and gas chromatography. JAA carried out the gene array. WEH gave the final approval of the version to be published.

## References

[B1] Simopoulos AP Evolutionary aspects of diet, the omega-6/omega-3 ratio and genetic variation: nutritional implications for chronic diseases Biomed Pharmacother 2006 60 502 507 10.1016/j.biopha.2006.07.080 17045449

[B2] Yashodhara BM Umakanth S Pappachan JM Bhat SK Kamath R Choo BH Omega-3 fatty acids: a comprehensive review of their role in health and disease Postgrad Med J 2009 85 84 90 10.1136/pgmj.2008.073338 19329703

[B3] Denys A Hichami A Khan NA n-3 PUFAs modulate T-cell activation via protein kinase C-alpha and -epsilon and the NF-kappaB signaling pathway J Lipid Res 2005 46 752 758 10.1194/jlr.M400444-JLR200 15627650

[B4] Siddiqui RA Jenski LJ Neff K Harvey K Kovacs RJ Stillwell W Docosahexaenoic acid induces apoptosis in Jurkat cells by a protein phosphatase-mediated process Biochim Biophys Acta 2001 1499 265 275 10.1016/S0167-4889(00)00128-2 11341974

[B5] Verlengia R Gorjao R Kanunfre CC Bordin S de Lima TM Curi R Effect of arachidonic acid on proliferation, cytokines production and pleiotropic genes expression in Jurkat cells--a comparison with oleic acid Life Sci 2003 73 2939 2951 10.1016/j.lfs.2003.04.003 14519443

[B6] Takahashi HK Cambiaghi TD Luchessi AD Hirabara SM Vinolo MA Newsholme P Curi R Activation of survival and apoptotic signaling pathways in lymphocytes exposed to palmitic acid J Cell Physiol 2011 in press 10.1002/jcp.22740 21437903

[B7] Costabile M Hii CS Melino M Easton C Ferrante A The immunomodulatory effects of novel beta-oxa, beta-thia, and gamma-thia polyunsaturated fatty acids on human T lymphocyte proliferation, cytokine production, and activation of protein kinase C and MAPKs J Immunol 2005 174 233 243 15611245 10.4049/jimmunol.174.1.233

[B8] Cury-Boaventura MF Gorjao R de Lima TM Newsholme P Curi R Comparative toxicity of oleic and linoleic acid on human lymphocytes Life Sci 2006 78 1448 1456 10.1016/j.lfs.2005.07.038 16236329

[B9] Vedin I Cederholm T Freund LY Basun H Garlind A Faxen IG Jonhagen ME Vessby B Wahlund LO Palmblad J Effects of docosahexaenoic acid-rich n-3 fatty acid supplementation on cytokine release from blood mononuclear leukocytes: the OmegAD study Am J Clin Nutr 2008 87 1616 1622 18541548 10.1093/ajcn/87.6.1616

[B10] Kim W Fan YY Barhoumi R Smith R McMurray DN Chapkin RS n-3 polyunsaturated fatty acids suppress the localization and activation of signaling proteins at the immunological synapse in murine CD4+ T cells by affecting lipid raft formation J Immunol 2008 181 6236 6243 18941214 10.4049/jimmunol.181.9.6236 PMC2597670

[B11] Bergamo P Luongo D Maurano F Rossi M Butterfat fatty acids differentially regulate growth and differentiation in Jurkat T-cells J Cell Biochem 2005 96 349 360 10.1002/jcb.20565 16052483

[B12] Peck MD Li Z Han T Wang W Jy W Ahn YS Ziboh VA Chu AJ Bourguignon LY Fatty acid unsaturation increases expression and capping of murine lymphocyte CD44 and CD45 Nutrition 1996 12 616 622 10.1016/S0899-9007(96)00177-3 8878171

[B13] Pompos LJ Fritsche KL Antigen-driven murine CD4+ T lymphocyte proliferation and interleukin-2 production are diminished by dietary (n-3) polyunsaturated fatty acids J Nutr 2002 132 3293 3300 12421842 10.1093/jn/132.11.3293

[B14] Shaikh SR Edidin M Immunosuppressive effects of polyunsaturated fatty acids on antigen presentation by human leukocyte antigen class I molecules J Lipid Res 2007 48 127 138 17074926 10.1194/jlr.M600365-JLR200

[B15] Lu X Yu H Ma Q Shen S Das UN Linoleic acid suppresses colorectal cancer cell growth by inducing oxidant stress and mitochondrial dysfunction Lipids Health Dis 2010 9 106 10.1186/1476-511X-9-106 20868498 PMC2954911

[B16] Yuan H Zhang X Huang X Lu Y Tang W Man Y Wang S Xi J Li J NADPH oxidase 2-derived reactive oxygen species mediate FFAs-induced dysfunction and apoptosis of beta-cells via JNK, p38 MAPK and p53 pathways PLoS One 2010 5 e15726 10.1371/journal.pone.0015726 21209957 PMC3012098

[B17] Nicoletti I Migliorati G Pagliacci MC Grignani F Riccardi C A rapid and simple method for measuring thymocyte apoptosis by propidium iodide staining and flow cytometry J Immunol Methods 1991 139 271 279 10.1016/0022-1759(91)90198-O 1710634

[B18] Kim JY Park HD Park E Chon JW Park YK Growth-inhibitory and proapoptotic effects of alpha-linolenic acid on estrogen-positive breast cancer cells Ann N Y Acad Sci 2009 1171 190 195 10.1111/j.1749-6632.2009.04897.x 19723055

[B19] Droge W Free radicals in the physiological control of cell function Physiol Rev 2002 82 47 95 11773609 10.1152/physrev.00018.2001

[B20] Razanamahefa L Prouff S Bardon S Stimulatory effect of arachidonic acid on T-47D human breast cancer cell growth is associated with enhancement of cyclin D1 mRNA expression Nutr Cancer 2000 38 274 280 10.1207/S15327914NC382_17 11525606

[B21] Chen ZY Istfan NW Docosahexaenoic acid, a major constituent of fish oil diets, prevents activation of cyclin-dependent kinases and S-phase entry by serum stimulation in HT-29 cells Prostaglandins Leukot Essent Fatty Acids 2001 64 67 73 10.1054/plef.2000.0239 11161587

[B22] Palakurthi SS Fluckiger R Aktas H Changolkar AK Shahsafaei A Harneit S Kilic E Halperin JA Inhibition of translation initiation mediates the anticancer effect of the n-3 polyunsaturated fatty acid eicosapentaenoic acid Cancer Res 2000 60 2919 2925 10850438

[B23] Cho HJ Kim EJ Lim SS Kim MK Sung MK Kim JS Park JH Trans-10, cis-12, not cis-9, trans-11, conjugated linoleic acid inhibits G1-S progression in HT-29 human colon cancer cells J Nutr 2006 136 893 898 16549447 10.1093/jn/136.4.893

[B24] Coussens LM Werb Z Inflammation and cancer Nature 2002 420 860 867 10.1038/nature01322 12490959 PMC2803035

[B25] Serhan CN Savill J Resolution of inflammation: the beginning programs the end Nat Immunol 2005 6 1191 1197 10.1038/ni1276 16369558

[B26] Matsunaga H Hokari R Kurihara C Okada Y Takebayashi K Okudaira K Watanabe C Komoto S Nakamura M Tsuzuki Y Kawaguchi A Nagao S Miura S Omega-3 polyunsaturated fatty acids ameliorate the severity of ileitis in the senescence accelerated mice (SAM)P1/Yit mice model Clin Exp Immunol 2009 158 325 333 10.1111/j.1365-2249.2009.04020.x 19793338 PMC2792829

[B27] Weylandt KH Krause LF Gomolka B Chiu CY Bilal S Nadolny A Waechter SF Fischer A Rothe M Kang JX Suppressed liver tumorigenesis in fat-1 mice with elevated omega-3 fatty acids is associated with increased omega-3 derived lipid mediators and reduced TNF-{alpha} Carcinogenesis 2011 in press 10.1093/carcin/bgr049 PMC3106436 21421544

[B28] Wilcox RA Wada DA Ziesmer SC Elsawa SF Comfere NI Dietz AB Novak AJ Witzig TE Feldman AL Pittelkow MR Ansell SM Monocytes promote tumor cell survival in T-cell lymphoproliferative disorders and are impaired in their ability to differentiate into mature dendritic cells Blood 2009 114 2936 2944 10.1182/blood-2009-05-220111 19671921 PMC2756204

[B29] Vaday GG Peehl DM Kadam PA Lawrence DM Expression of CCL5 (RANTES) and CCR5 in prostate cancer Prostate 2006 66 124 134 10.1002/pros.20306 16161154

[B30] Yaal-Hahoshen N Shina S Leider-Trejo L Barnea I Shabtai EL Azenshtein E Greenberg I Keydar I Ben-Baruch A The chemokine CCL5 as a potential prognostic factor predicting disease progression in stage II breast cancer patients Clin Cancer Res 2006 12 4474 4480 10.1158/1078-0432.CCR-06-0074 16899591

[B31] Aldinucci D Lorenzon D Cattaruzza L Pinto A Gloghini A Carbone A Colombatti A Expression of CCR5 receptors on Reed-Sternberg cells and Hodgkin lymphoma cell lines: involvement of CCL5/Rantes in tumor cell growth and microenvironmental interactions Int J Cancer 2008 122 769 776 10.1002/ijc.23119 17935139

[B32] Lapteva N Huang XF CCL5 as an adjuvant for cancer immunotherapy Expert Opin Biol Ther 2010 10 725 733 10.1517/14712591003657128 20233026

[B33] Xie K Interleukin-8 and human cancer biology Cytokine Growth Factor Rev 2001 12 375 391 10.1016/S1359-6101(01)00016-8 11544106

[B34] Humke EW Shriver SK Starovasnik MA Fairbrother WJ Dixit VM ICEBERG: a novel inhibitor of interleukin-1beta generation Cell 2000 103 99 111 10.1016/S0092-8674(00)00108-2 11051551

[B35] Garcia-Zepeda EA Rothenberg ME Ownbey RT Celestin J Leder P Luster AD Human eotaxin is a specific chemoattractant for eosinophil cells and provides a new mechanism to explain tissue eosinophilia Nat Med 1996 2 449 456 10.1038/nm0496-449 8597956

[B36] Salcedo R Young HA Ponce ML Ward JM Kleinman HK Murphy WJ Oppenheim JJ Eotaxin (CCL11) induces in vivo angiogenic responses by human CCR3+ endothelial cells J Immunol 2001 166 7571 7578 11390513 10.4049/jimmunol.166.12.7571

